# PRMT5 enhances tumorigenicity and glycolysis in pancreatic cancer via the FBW7/cMyc axis

**DOI:** 10.1186/s12964-019-0344-4

**Published:** 2019-03-29

**Authors:** Yi Qin, Qiangsheng Hu, Jin Xu, Shunrong Ji, Weixing Dai, Wensheng Liu, Wenyan Xu, Qiqing Sun, Zheng Zhang, Quanxing Ni, Bo Zhang, Xianjun Yu, Xiaowu Xu

**Affiliations:** 1 Department of Pancreatic Surgery, Fudan University Shanghai Cancer Center, Shanghai, 200032 China; 2 Department of Oncology, Shanghai Medical College, Fudan University, Shanghai, 200032 China; 3 Shanghai Pancreatic Cancer Institute, Shanghai, 200032 China; 4 Pancreatic Cancer Institute, Fudan University, Shanghai, 200032 China; 5 Cancer Research Institute, Fudan University Shanghai Cancer Center, Shanghai, 200032 China

**Keywords:** PRMT5, Aerobic glycolysis, cMyc, FBW7, Pancreatic cancer

## Abstract

**Background:**

The epigenetic factor protein arginine methyltransferase 5 (PRMT5) has been reported to play vital roles in a wide range of cellular processes, such as gene transcription, genomic organization, differentiation and cell cycle control. However, its role in pancreatic cancer remains unclear. Our study aimed to investigate the roles of PRMT5 in pancreatic cancer prognosis and progression and to explore the underlying molecular mechanism.

**Methods:**

Real-time PCR, immunohistochemistry and analysis of a dataset from The Cancer Genome Atlas (TCGA) were performed to study the expression of PRMT5 at the mRNA and protein levels in pancreatic cancer. Cell proliferation assays, including cell viability, colony formation ability and subcutaneous mouse model assays, were utilized to confirm the role of PRMT5 in cell proliferation and tumorigenesis. A Seahorse extracellular flux analyzer, a glucose uptake kit, a lactate level measurement kit and the measurement of ^18^F-FDG (fluorodeoxyglucose) uptake by PET/CT (positron emission tomography/computed tomography) imaging were used to verify the role of PRMT5 in aerobic glycolysis, which sustains cell proliferation. The regulatory effect of PRMT5 on cMyc, a master regulator of oncogenesis and aerobic glycolysis, was explored by quantitative PCR and protein stability measurements.

**Results:**

PRMT5 expression was significantly upregulated in pancreatic cancer tissues compared with that in adjacent normal tissues. Clinically, elevated expression of PRMT5 was positively correlated with worse overall survival in pancreatic cancer patients. Silencing PRMT5 expression inhibited the proliferation of pancreatic cancer cells both in vitro and in vivo. Moreover, PRMT5 regulated aerobic glycolysis in vitro in cell lines, in vivo in pancreatic cancer patients and in a xenograft mouse model used to measure 18F-FDG uptake. We found that mechanistically, PRMT5 posttranslationally regulated cMyc stability via F-box/WD repeat-containing protein 7 (FBW7), an E3 ubiquitin ligase that controls cMyc degradation. Moreover, PRMT5 epigenetically regulated the expression of FBW7 in pancreatic cancer cells.

**Conclusions:**

The present study demonstrated that PRMT5 epigenetically silenced the expression of the tumor suppressor FBW7, leading to increased cMyc levels and the subsequent enhancement of the proliferation of and aerobic glycolysis in pancreatic cancer cells. The PRMT5/FBW7/cMyc axis could be a potential therapeutic target for the treatment of pancreatic cancer.

**Electronic supplementary material:**

The online version of this article (10.1186/s12964-019-0344-4) contains supplementary material, which is available to authorized users.

## Background

Pancreatic cancer is a devastating disease with an extremely high mortality rate. Although significant progress has been made in past decades in the diagnosis and treatment of pancreatic cancer, the overall 5-year survival rate of patients remains steady, at approximately 6% [1, 2]. The high proliferation and metastasis capacity of pancreatic cancer primarily account for the poor prognosis of the disease. Thus, there is an urgent need for a better understanding of the molecular basis underlying these malignant properties of pancreatic cancer [3].

Posttranslational methylation of histone lysine (K) or arginine (R) residues can regulate chromatin structures that lead to changes in gene expression, participate in the modulation of cellular processes and are often deregulated during cancer pathogenesis [4, 5]. Therefore, targeting enzymes that add methyl groups to or remove methyl groups from K or R residues in substrates is an attractive strategy for anticancer drug design [6]. In the past two decades, much emphasis has been placed on histone lysine methylation, and the discovery and development of histone lysine methylation modifiers, including histone lysine methyltransferases (KMTs) and histone lysine demethylases (KDMs), have progressed rapidly [7, 8]. A series of highly selective compounds that target KMTs and KDMs have been reported and display extensive preclinical efficacy [9]. However, the importance of arginine methylation catalyzed by protein arginine methyltransferases (PRMTs) in oncogenesis and cancer progression is only recently becoming apparent [10, 11]. Of special interest, the expression of PRMT5 has been reported to be correlated with poor prognosis in patients with different cancer types [12]. For example, PRMT5 has been reported to be frequently overexpressed and correlated with poor outcomes in lung cancer [13]. In hepatocellular carcinoma (HCC), upregulation of PRMT5 correlated with a worse prognosis, and PRMT5 could regulate the proliferation of HCC cells in vitro [14]. In breast cancer, PRMT5 functions as a critical regulator of stem cell traits and is a promising therapeutic target [15]. One of the most recent studies linked PRMT5 to genetic variations in cancer cell tumorigenicity. Heterozygous deletion of p16/CDKN2A is prevalent in cancer, and these mutations commonly involve a codeletion of adjacent genes, including methylthioadenosine phosphorylase (MTAP). shRNA screening strategies identified PRMT5 as a vulnerable enzyme in cells with CDKN2A deletion [16, 17]. In pancreatic cancer, CDKN2A is one of the most frequently altered genes, and its inactivation caused by loss of heterozygosity, homozygous deletion or promoter silencing has been observed in 98% of pancreatic cancer cases [18]. However, the importance of the major downstream target of CDKN2A deletion, PRMT5, in pancreatic cancer has seldom been discussed. Therefore, it is of vital importance to uncover the roles of PRMT5 in pancreatic cancer with an aim to identify novel prognostic and treatment targets.

PRMT5 regulates the expression of a wide spectrum of target genes via modification of the chromatin structure or transcriptional machinery. Specifically, PRMT5 can catalyze the methylation of arginine 8 on histone H3 (H3R8) and arginine 3 on histone H4 (H4R3), resulting in the silencing of cell cycle genes such as cyclin E1 (CCNE1) [19]. In addition, certain tumor suppressors, such as the metastasis inhibition factor Nm23, can be epigenetically silenced by PRMT5 [20]. Moreover, components of the transcription machinery are substrates for PRMT5-catalyzed methylation modifications. For example, the transcription elongation factor SPT5 can be methylated by PRMT5, and this methylation modification can regulate the interaction of SPT5 with RNA polymerase II, leading to transcriptional elongation [21]. Thus, PRMT5 primarily functions as a tumor-promoting factor. It is well acknowledged that cells undergoing uncontrolled proliferation require a constant supply of nutrients for the macromolecule synthesis and generation of corresponding energy in the form of adenosine triphosphate (ATP) that are required. In solid tumors, due to limitations in oxygen and the nutrient supply caused by abnormal blood vessels, tumor cells must reprogram their metabolic pattern to sustain uncontrolled proliferation. The best-characterized type of metabolic reprogramming is aerobic glycolysis, which supports cell proliferation under hypoxic conditions [22, 23]. Some epigenetic factors have been reported to regulate aerobic glycolysis to support tumorigenesis. G9a, which catalyzes H3K9 methylation, has been reported to epigenetically silence fructose-1,6-bisphosphatase 1 (FBP1), leading to enhanced aerobic glycolysis in breast cancer [24]. In our previous studies, we demonstrated that histone lysine-specific demethylase 1 (LSD1) was a positive regulator of aerobic glycolysis in pancreatic cancer [25]. However, the relevant roles of PRMT5 in aerobic glycolysis that sustain pancreatic cancer tumorigenicity have seldom been discussed.

In the present study, our results demonstrated that PRMT5 was an unfavorable prognostic factor for pancreatic cancer. The results of in vitro cell line studies and in vivo subcutaneous mouse model experiments demonstrated that PRMT5 could regulate the tumorigenicity of pancreatic cancer cells. In-depth mechanistic explorations indicated that PRMT5 could regulate aerobic glycolysis via the regulation of cMyc stability by epigenetically silencing the expression of FBW7, a ubiquitin ligase and tumor suppressor with decreased expression in cancer cells [26, 27]. Collectively, the results of our present study uncovered a novel PRMT5/FBW7/cMyc axis in pancreatic cancer, and targeting this axis might be a strategy for the treatment of pancreatic cancer.

## Material and methods

### Cell lines and chemical reagents

The human pancreatic cancer cell lines PANC-1, MIA PaCa-2, Capan-1, BxPC-3 and SW1990 were obtained from the American Type Culture Collection (ATCC, Manassas, VA, USA), and the cell lines were cultured according to standard protocols provided by ATCC. The human pancreatic ductal epithelial cell line HPDE was kindly provided by Professor Min Li at Ohio State University and routinely cultured in keratinocyte serum-free (KSF) medium supplemented with epidermal growth factor and bovine pituitary extract (Life Technologies, Inc., Grand Island, NY).

### Protein extraction and western blot analysis

Cells were washed twice with ice-cold PBS, harvested and lysed for 10 min in RIPA buffer (20 mM Tris/HCl, pH 8.0;, 150 mM NaCl; 20 μM EDTA; 1% NP40; and 10% glycerol) supplemented with protease and phosphatase inhibitors. For complete lysis, the lysates were sonicated and then centrifuged at 12000 rpm for 20 min at 4 °C. The concentrations of the lysates were determined by a BCA protein assay reagent kit (Pierce, Waltham, MA, USA). A total of 20 μg of total protein lysate was subjected to electrophoresis on denaturing 10% SDS-polyacrylamide gels and was then transferred to a membrane for subsequent blotting with specific antibodies. Antibodies against PRMT5, HIF1α and cMyc were purchased from Abcam (Cambridge, UK). Antibodies against FBW7 were obtained from Bethyl Laboratories (Montgomery, TX, USA).

### RNA extraction and quantitative PCR

Total RNA was extracted by using Invitrogen TRIzol reagent. To obtain cDNA, a TaKaRa PrimeScript RT kit (TaKaRa, Dalian, China) was used. The expression status of the designated genes was determined by real-time PCR using an ABI 7900HT Real-Time PCR system (Applied Biosystems, USA). To examine the mRNA expression status of PRMT5 in pancreatic cancer patients, 30 paired samples from patients diagnosed with PDAC were used. Total RNA was extracted from samples stored in RNAlater. β-Actin was used as the loading control. All reactions were performed in triplicate. The primer sequences are listed in Additional file 1: Table S1.

### Plasmids

To silence PRMT5 expression, the pLKO.1 TRC cloning vector (Addgene plasmid 10,878, Watertown, MA, USA) was used. The 21 bp targets against PRMT5 were GGCTCAAGCCACCAATCTATG and CCCATCCTCTTCCCTATTAAG, respectively. Scrambled shRNA or the Scr vector (Addgene plasmid 1864) was used as a knockdown control vector. The pCDH-CMV-MCS-EF1-Puro vector (System Biosciences, Palo Alto, CA, USA) was used to overexpress PRMT5, FBW7, and the corresponding mutants; empty vector (EV) was used as the control.

### Cell proliferation assay

To determine the roles of PRMT5 in pancreatic cancer cell proliferation, we performed in vitro cell viability assays by using a Cell Counting Kit-8 (CCK-8, Dojindo, Japan) and colony formation assays according to previous reports [28].

### Tumorigenesis study

BALB/c-nu mice (5–6 weeks of age, 18–20 g, Shanghai SLAC Laboratory Animal Co., Ltd.) were housed in sterile filter-capped cages. A total of 4 × 10^6^ PRMT5-silenced or the corresponding control SW1990 cells were suspended in 100 μl of phosphate-buffered saline and injected subcutaneously into the back of the mice. The tumor size was measured weekly with calipers beginning at the formation of palpable tumors. The tumor volume was calculated by the following formula: length × width^2^ × 0.5^2^. Six weeks after implantation, the mice were euthanized, and the tumors were surgically dissected. Samples were then processed for histopathological examination. All animal experiments were performed according to the Guidelines for the Care and Use of Laboratory Animals and were approved by the IACUC of Fudan University.

### In vitro and in vivo glycolysis measurement

To confirm the role of PRMT5 in aerobic glycolysis, we performed glycolysis measurements using a Seahorse Extracellular Flux Analyzer, an Abcam Glucose Uptake Assay Kit and a Biovision Lactate Colorimetric Assay Kit according to the protocols supplied by the manufacturers. In vivo glucose utilization by subcutaneous tumors was measured by using microPET/CT imaging to assess 18F-FDG uptake in xenograft tumors, according to our previous reports. Briefly, each tumor-bearing mouse was injected with 11.1 MBq (300 μCi) of 18F-FDG via the tail vein. Scanning started 1 h after injection. Animals were anesthetized with isoflurane during the scanning period. The images were reconstructed using a three-dimensional ordered subset expectation maximization (OSEM3D)/maximum a posteriori algorithm. Inveon Research Workplace was used to obtain the percentage injected dose per gram (%ID/g) and the SUVs (standardized uptake value). The SUVmax was calculated [29].

### Analysis of tumor glucose uptake in pancreatic cancer patients

To assess the correlation between PRMT5 and glucose uptake in pancreatic cancer patients, we examined the SUVmax in pancreatic cancer patients via PET/CT imaging, a technique that measures glucose uptake via glycolysis by assessing 18F-FDG uptake. The SUVmax was obtained and calculated according to our previous reports. Briefly, ^18^F-FDG was automatically made in a cyclotron (Siemens CTI RDS Eclipse ST) using an Explora FDG4 module. Patients were fasted for more than 6 h. Scanning started 1 h after intravenous injection of the tracer (7.4 MBq/kg). Images were acquired on a Siemens biograph 16HR PET/CT scanner with a transaxial intrinsic spatial resolution of 4.1 mm. CT scanning was initiated from the proximal thighs to the head at 120 kV and 80–250 mA with a pitch of 3.6 and a rotation time of 0.5 s. Image interpretation was carried out on a multimodality computer platform (Syngo, Siemens). Metabolic activity was quantified using the SUVs normalized to the body weights of the patients, and the SUVmax for each lesion was calculated [29].

### Tissue specimens and immunohistochemistry (IHC)

The clinical pancreatic tumor samples used in the manuscript were obtained from patients diagnosed with pancreatic cancer at Fudan University Shanghai Cancer Center (FDUSCC), with approval from the Institutional Research Ethics Committee. Immunohistochemical staining of designated factors was performed according to our previous reports [30]. Paratumor and tumor samples from 30 patients were used to assess the expression status of PRMT5 by immunohistochemical staining. In addition, samples from 55 patients diagnosed with pancreatic adenocarcinoma and matched with follow-up information were used to analyze the impact of PRMT5 on prognosis by immunohistochemical staining. The clinicopathological features of the 55 patients are listed in Additional file 1: Table S2. Antibodies used for immunohistochemical staining of PRMT5 and Ki67 were purchased from Abcam. The anti-FBW7 IHC antibody for the detection of FBW7 was obtained from Bethyl Laboratories. The antibodies against PRMT5, Ki67 and FBW7 were used at dilutions of 1:50, 1:200 and 1:100, respectively.

### Promoter luciferase activity measurement

To confirm the impact of PRMT5 on FBW7 promoter activity, a dual-luciferase assay was performed. In brief, the promoter region of FBW7 covering the region from − 2000 to 200 base pairs was amplified from pancreatic cancer cells and ligated into the pGL3-Basic vector (Promega, Madison, WI, USA). PRMT5, pGL3-FBW7 and the control Renilla luciferase vector pRL-TK were cotransfected into cells, and luciferase activity was measured by using a Promega Dual Luciferase Reporter Assay System.

### Chromatin immunoprecipitation (ChIP)

ChIP assays were performed by using a Millipore EZ ChIP Kit. An antibody against PRMT5 (MERCK, Darmstadt, Germany) was used for ChIP. ChIP-grade anti-histone H3 (acetyl K9), anti-histone H3 (tri methyl K9) and anti-histone 4 (symmetric di methyl R3) antibodies were purchased from Abcam. The primer sequences that covered the FBW7 promoter region with hypermethylation were F: 5′-CCCGGGAGAAGTGGCCCTGGACG-3′ and R: 5′-TTCGGACTGAAGCGGCAGCTGCGGA-3’ [31]. A quantitative ChIP assay was performed according to previous reports. DNA extracted from saved input samples was quantitated in parallel (Ct [Input]) and adjusted to a relative expression of 100% using the following equation: Adjusted Ct [Input]  =  Ct [Input] − 4.322. (log2(5%)  =   − 4.322.) The IP results with normal IgG or specific antibody (Ct [IP]) were then used to calculate the relative nonspecific background and specific occupancy using the following equation: 2^(Adjusted Ct [Input] − Ct [IP]) * 100% [32].

### TCGA data analysis

TCGA-PAAD RNA expression data (Level 3) for pancreatic cancer patients obtained by RNA-seq by expectation-maximization was downloaded from the Cancer Genomics Brower of the University of California, Santa Cruz (UCSC) (https://genome-cancer.ucsc.edu/). In total, 177 primary pancreatic cancer samples from patients with detailed expression data were selected from the updated TCGA database according to the parameters mentioned.

### Statistical analysis

Statistical analyses were performed by SPSS software (version 17.0, IBM Corp., Armonk, NY, USA) using independent paired t-tests (two-tailed) or one-way analysis of variance (ANOVA). Differences were considered significant at *, *P* < 0.05; **, *P* < 0.01; and ***, *P* < 0.001.

## Results

### PRMT5 expression is upregulated and indicates a worse prognosis in pancreatic cancer patients

**Fig. 1:**
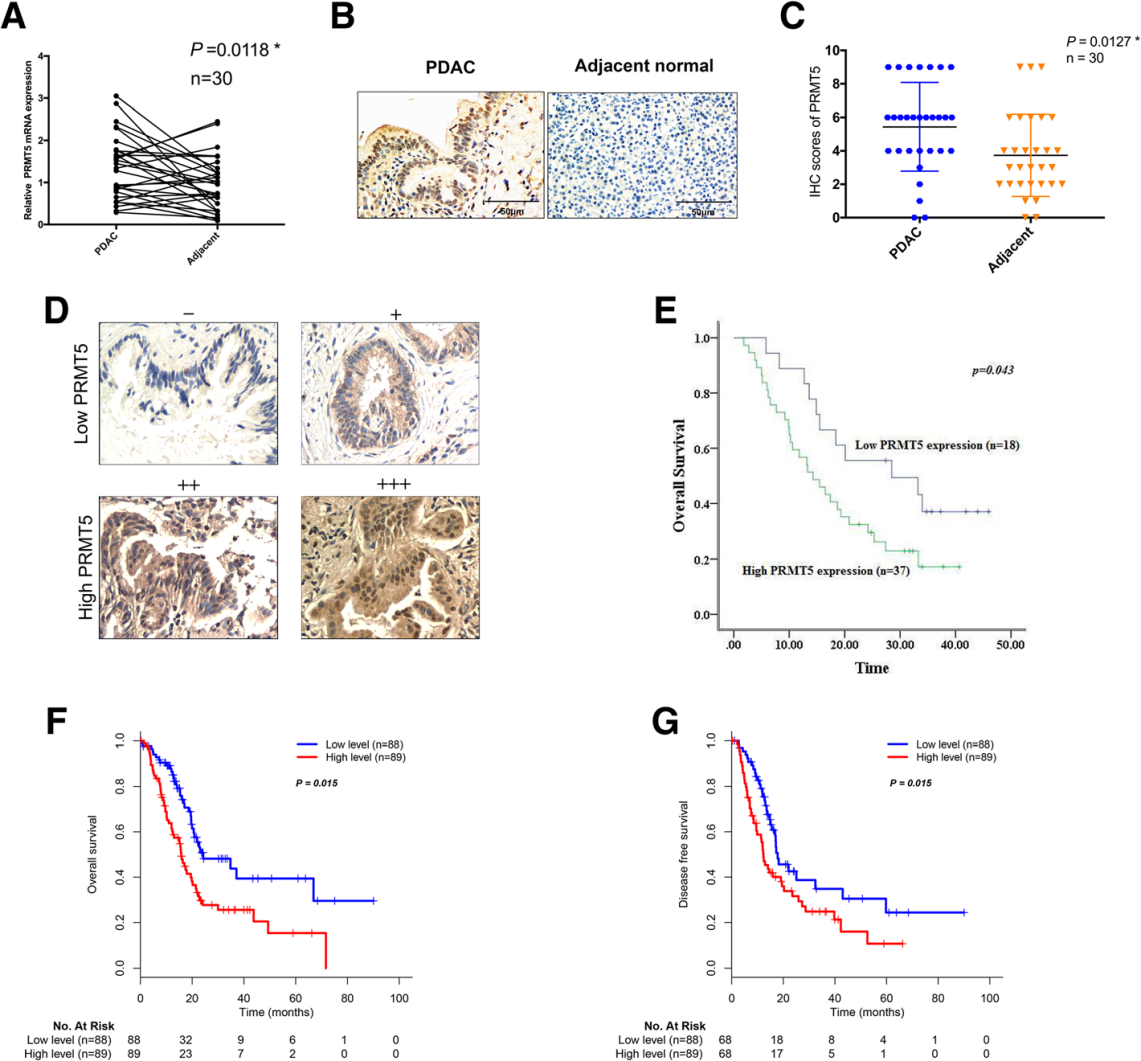
PRMT5 expression is upregulated and indicates a worse prognosis in pancreatic cancer patients **a.** PRMT5 expression was upregulated in PDAC tumor samples compared with that in normal adjacent samples, as indicated by real-time PCR and paired t-test analysis. **b.** Immunohistochemical staining of PRMT5 in PDAC tumor samples and paired adjacent normal tissues. **c.** PRMT5 protein levels were increased in tumor samples, as reflected by IHC analysis. **d.** IHC scoring of PRMT5 expression in pancreatic cancer tissues, which were divided into the low PRMT5 expression and high PRMT5 expression subgroups. **e.** Patients in the FDUSCC cohort with high PRMT5 expression had shorter overall survival times than those with low PRMT5 expression. **f.** PRMT5 expression in the TCGA-PAAD RNA-seq dataset. Analysis results demonstrated that high PRMT5 expression predicts a shorter OS time. **g.** Patients with high PRMT5 expression had shorter DFS times than patients with low PRMT5 expression

The importance of PRMT5 expression in pancreatic cancer has seldom been discussed. First, by using quantitative real-time PCR and a paired t-test statistical analysis, we demonstrated that PRMT5 expression was higher in pancreatic adenocarcinoma (PDAC) tumor samples than in adjacent samples (Fig. 1a). Next, we performed immunohistochemical staining to measure the expression status of PRMT5 in pancreatic cancer patients, and our results demonstrated that PRMT5 expression was significantly higher in PDAC tumor samples than in adjacent normal tissues (Fig. 1b and c). Subsequently, we investigated the ability of PRMT5 expression to predict overall survival in patients with pancreatic cancer. The expression scores were defined as -, +, ++, and +++ (Fig. 1d). Overall survival analysis demonstrated that patients with higher PRMT5 expression displayed worse prognoses than patients with lower PRMT5 expression (Fig. 1e). The correlations between PRMT5 expression and clinicopathological features are shown in Additional file 1: Table S2. Finally, we validated the results by using the TCGA pancreatic cancer patient cohort. In the patients included in the TCGA database, PRMT5 was an unfavorable prognostic marker, and patients with higher PRMT5 expression displayed shorter overall survival (OS) and disease-free survival (DFS) times than those with lower PRMT5 expression (Fig. 1f and g). The correlations between PRMT5 expression and the clinicopathological features of the TCGA cohort patients are shown in Additional file 1: Table S3. Thus, PRMT5 expression is elevated in pancreatic cancer patients and indicates a worse prognosis for pancreatic cancer patients.

### PRMT5 regulates the proliferation and tumorigenicity of pancreatic cancer cells

**Fig. 2:**
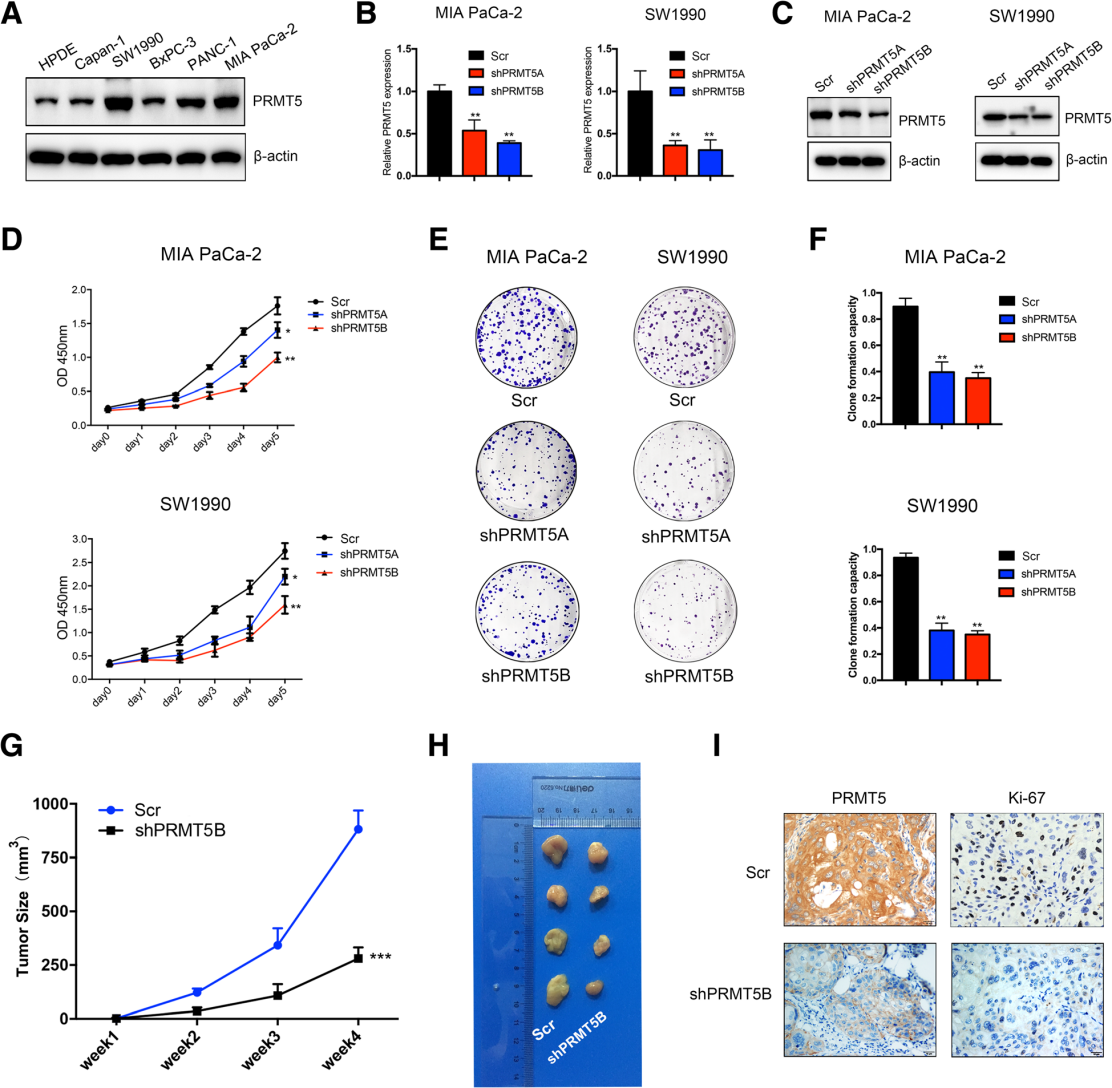
PRMT5 regulates the proliferation and tumorigenicity of pancreatic cancer cells **a.** PRMT5 expression in pancreatic cancer cell lines. **b.** Quantitative real-time results confirmed the silencing efficiency of shRNAs targeting PRMT5 in MIA PaCa-2 and SW1990 cells. **c.** Immunoblotting with an anti-PRMT5 antibody further confirmed that PRMT5 was efficiently silenced in MIA PaCa-2 and SW1990 cells. **d.** Silencing of PRMT5 decreased the viability of MIA PaCa-2 and SW1990 cells (*n* = 3, *p < 0.05* and *p < 0.01* for the shPRMT5A and shPRMT5B groups, respectively). **e-f.** Silencing of PRMT5 inhibited the colony formation capacity of MIA PaCa-2 and SW1990 cells (n = 3, *p < 0.01*). **g-h.** The subcutaneous xenograft mouse model showed that knockdown of PRMT5 decreased the tumor formation capacity of SW1990 cells (*n* = 4, *p < 0.001*). **i.** Representative images of immunohistochemical staining for Ki-67 and PRMT5

To confirm the roles of PRMT5 in tumorigenicity, we performed in vitro proliferation assays. First, we examined the expression status of PRMT5 in pancreatic cancer cell lines, including HPDE, Capan-1, PANC-1, BxPC-3, MIA PaCa-2 and SW1990. The results of western blots with the anti-PRMT5 antibody demonstrated that the PRMT5 level was higher in MIA PaCa-2 and SW1990 cells, while HPDE and Capan-1 cells displayed lower protein levels of PRMT5 (Fig. 2a). Next, we silenced PRMT5 in MIA PaCa-2 and SW1990 cells by using lentivirus-mediated shRNA transfection. The two shRNA targets against PRMT5 were designated shPRMT5A and shPRMT5B, while the scrambled shRNA transfected into control cells was designated Scr. The silencing efficiency of the shRNAs was confirmed by using quantitative real-time PCR and western blot analyses (Fig. 2b and c). In vitro cell proliferation analysis with the CCK-8 assay demonstrated that silencing PRMT5 expression decreased the viability of MIA PaCa-2 and SW1990 cells (Fig. 2d). The colony formation assay results suggested that silencing PRMT5 expression decreased the colony formation capacity of MIA PaCa-2 and SW1990 cells (Fig. 2e and f). Finally, through subcutaneous injection of PRMT5-silenced SW1990 cells into nude mice, we demonstrated that silencing PRMT5 decreased the tumor formation capacity of cells compared with that of the corresponding Scr cells (Fig. 2g and h). The Ki67 staining results further confirmed the roles of PRMT5 in promoting tumorigenicity (Fig. 2i). Collectively, these in vitro and in vivo results demonstrated that PRMT5 could regulate proliferation and tumorigenicity in pancreatic cancer cells.

### PRMT5 regulates aerobic glycolysis in vitro and in vivo

**Fig. 3:**
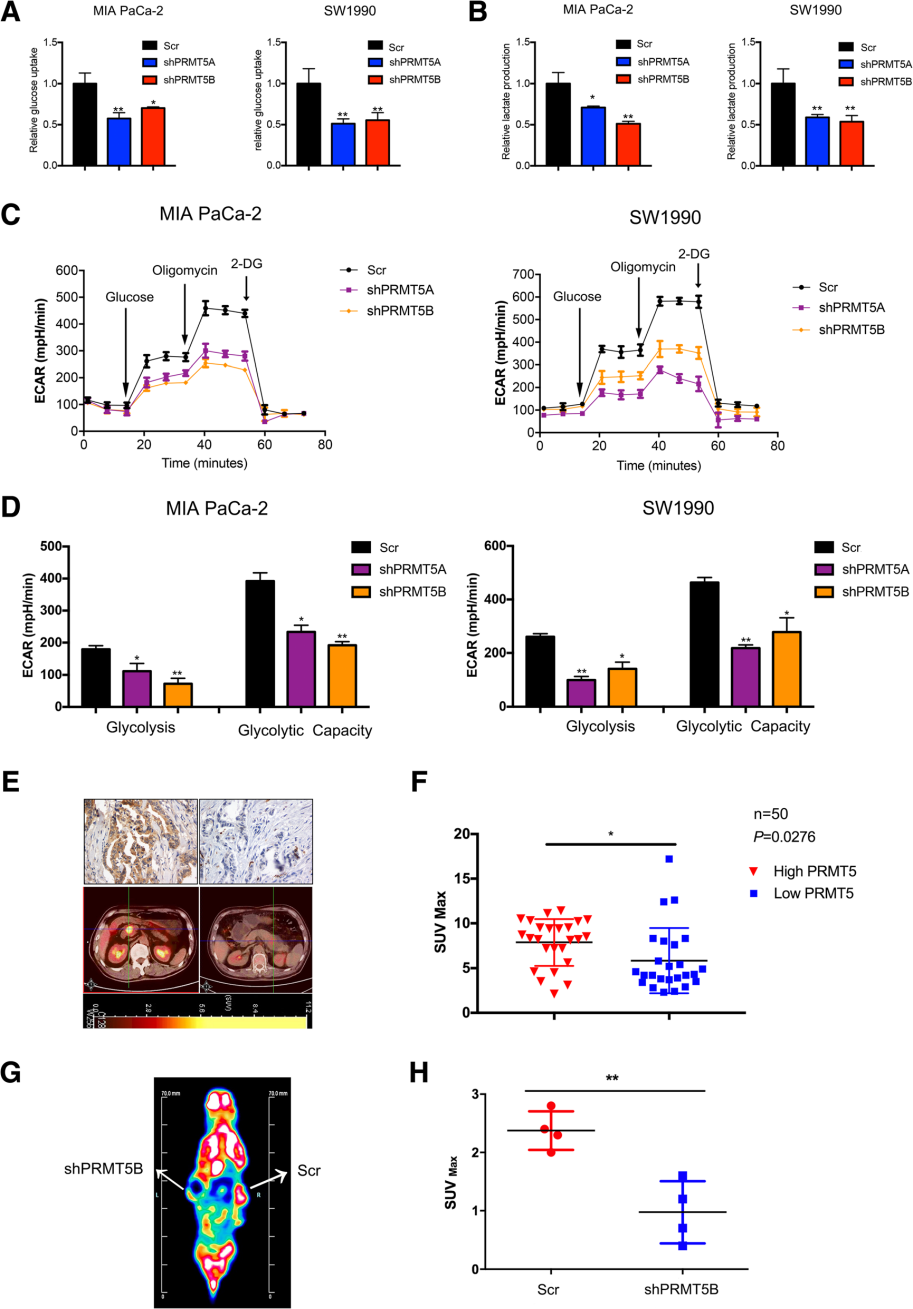
PRMT5 regulates aerobic glycolysis in vitro and in vivo **a.** The glucose uptake assay results suggested that PRMT5 knockdown decreased the glucose intake capacity of MIA PaCa-2 and SW1990 cells. **b.** The lactate level assay results indicated a decrease in lactate levels when PRMT5 was silenced in MIA PaCa-2 and SW1990 cells. **c-d.** A Seahorse extracellular flux analyzer was used to measure the ECAR, and the results indicated that decreased PRMT5 expression resulted in increased glycolysis in and glycolytic capacity of pancreatic cancer cells. **e.** Representative PRMT5 staining and 18F-FDG-PET/CT images for the indicated patients. **f.** Patients with high PRMT5 expression exhibited high levels of SUVmax values obtained by 18F-FDG-PET/CT imaging. **g.** Representative micro-PET/CT image of the subcutaneous xenograft mouse model. **h.** PRMT5 knockdown decreased 18F-FDG uptake by subcutaneous tumors, as reflected by the SUVmax value, reinforcing the roles of PRMT5 in promoting glycolysis in vivo

As noted, cancer cells are dependent on aerobic glycolysis for the supply of nutrients and energy. Thus, we asked whether PRMT5 could regulate glucose metabolism in pancreatic cancer cells. Compared with the corresponding control cells, PRMT5-silenced MIA PaCa-2 and SW1990 cells exhibited decreased glucose intake (Fig. 3a). In the process of aerobic glycolysis, cancer cells utilize glucose to generate lactate, which can be measured by lactate production assays. PRMT5-silenced cells exhibited a reduction in lactate levels (Fig. 3b). Next, to further confirm the role of PRMT5 in aerobic glycolysis, we performed ECAR measurements using a Seahorse extracellular flux analyzer, and these results further confirmed that the decreased PRMT5 expression in MIA PaCa-2 and SW1990 cells inhibited glycolysis in and the glycolytic capacity of these cells (Fig. 3c and d). Subsequently, we assessed the potential roles of PRMT5 in the regulation of aerobic glycolysis in vivo. PET/CT image scanning is a technique that can assess aerobic glycolysis in pancreatic cancer patients. Cancer cells with enhanced glycolytic capacity can absorb 18F-labeled FDG, and the accumulation of 18F-FDG in the body can be measured by PET/CT scanning equipment and calculated as the SUVmax value. Thus, we measured the expression status of PRMT5 by immunohistochemical staining and examined its correlation with the SUVmax obtained by PET/CT imaging, which reflects glucose uptake in pancreatic cancer patients. Our results demonstrated that patients with higher PRMT5 expression exhibited elevated 18F-FDG uptake (Fig. 3e and f). By using microPET/CT scanning, we observed that silencing PRMT5 expression attenuated 18F-FDG uptake by subcutaneous tumors in the mouse model, reconfirming the roles of PRMT5 in aerobic glycolysis in vivo (Fig. 3g and h). Thus, PRMT5 could potentially regulate aerobic glycolysis both in vitro and in vivo in pancreatic cancer.

### PRMT5 regulates the stability of cMyc

**Fig. 4:**
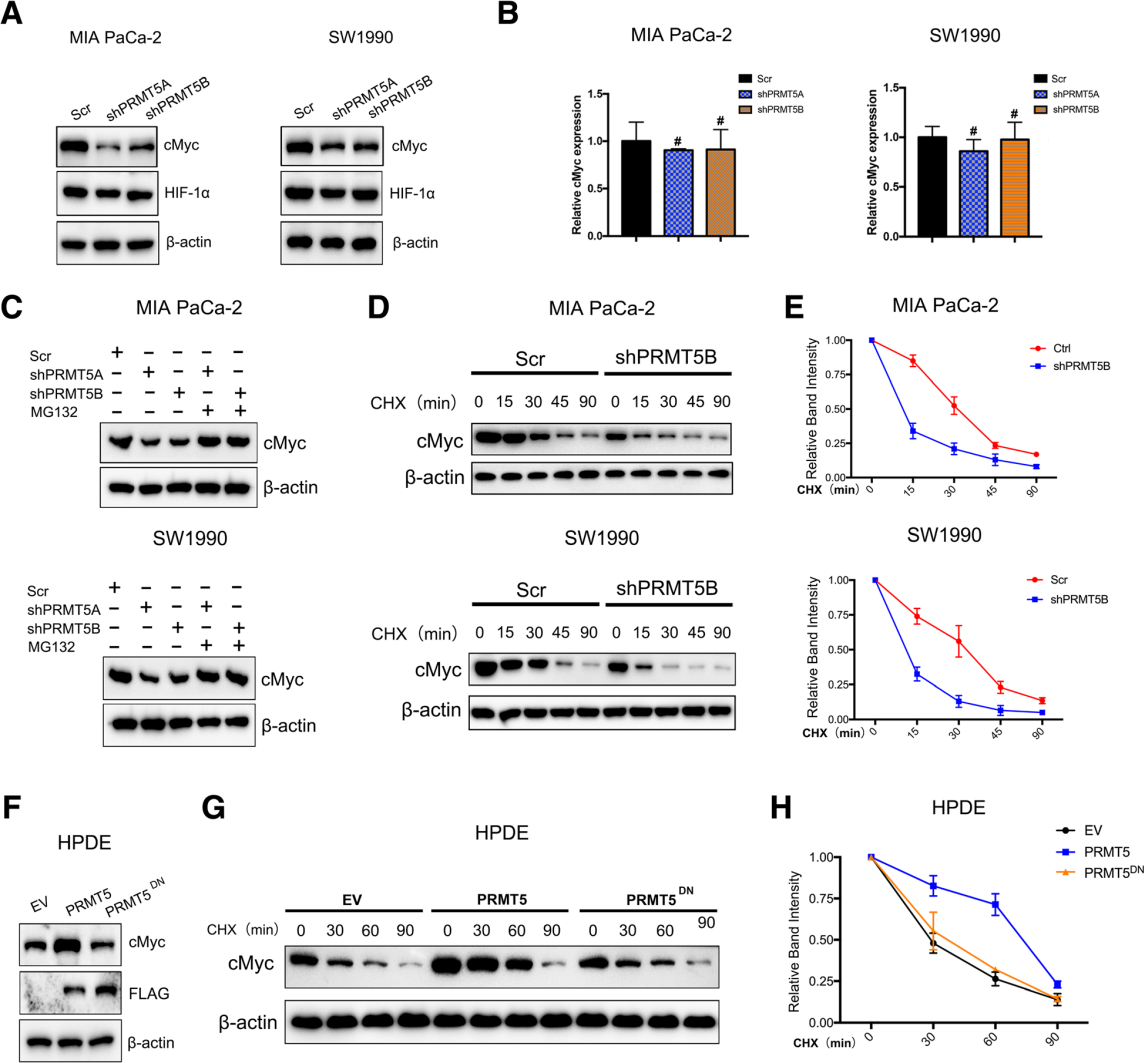
PRMT5 regulates cMyc at the posttranslational level **a.** Decreased PRMT5 expression resulted in a reduction in the cMyc protein level but had little impact on the HIF1α protein level. b**.** Knockdown of PRMT5 had a subtle impact on cMyc mRNA levels. **c.** We treated PRMT5-silenced pancreatic cancer cells with the proteasome inhibitor MG132 and measured the level of cMyc. The western blot analysis results demonstrated that MG132 treatment reversed the down-regulation of cMyc caused by PRMT5 knockdown, suggesting that PRMT5 regulates cMyc protein stability. **d.** PRMT5-silenced MIA PaCa-2 and SW1990 cells and the corresponding control cells were treated with CHX for the indicated durations, and the protein levels of cMyc were determined by western blotting. **e.** PRMT5 knockdown decreased the protein stability of cMyc in MIA PaCa-2 and SW1990 cells. **f**. In HPDE cells, overexpression of PRMT5 increased cMyc expression at the protein level, while the dominant-negative mutant of PRMT5 (PRMT5^DN^) had no such effect. **g-h.** Overexpressing PRMT5 in HPDE cells could stabilize cMyc, while PRMT5^DN^ had no such effect

cMyc and HIF1α have been reported to be decisive regulators of tumorigenesis and aerobic glycolysis; thus, we asked whether PRMT5 could regulate the expression of cMyc or HIF1α. PRMT5-silenced MIA PaCa-2 and SW1990 cells exhibited no significant change in the HIF1α protein level, but the protein level of cMyc was decreased (Fig. 4a). Interestingly, we observed no significant reduction in the mRNA level of cMyc, indicating that PRMT5 might regulate cMyc at the posttranslational level (Fig. 4b). We treated PRMT5-silenced pancreatic cancer cells with the proteasome inhibitor MG132, and the western blotting results demonstrated that MG132 could attenuate the decrease in the cMyc protein level, further confirming the hypothesis that PRMT5 could regulate cMyc at the posttranslational level (Fig. 4c). We measured the protein stability of cMyc in PRMT5-silenced pancreatic cancer cells after treatment with the protein synthesis inhibitor cycloheximide (CHX) at 100 μM, which could allow the protein stability of cMyc to be assessed. We observed that knockdown of PRMT5 decreased cMyc protein stability in MIA PaCa-2 and SW1990 cells (Fig. 4d and e). Furthermore, introduction of PRMT5 into HPDE cells could enhance the protein levels of cMyc, but introduction of the PRMT5 methyltransferase-dead mutants PRMT5^G367A/R368A^ or PRMT5^DN^ had little impact on cMyc protein levels (Fig. 4f). Subsequent measurements of cMyc protein stability demonstrated that PRMT5 but not PRMT5^DN^ could stabilize cMyc at the protein level (Fig. 4g and h). These results confirmed that PRMT5 could regulate cMyc protein stability and that its impact on cMyc protein stability was dependent on PRMT5 methyltransferase activity.

### PRMT5-mediated epigenetic silencing of FBW7 leads to increased cMyc levels

**Fig. 5:**
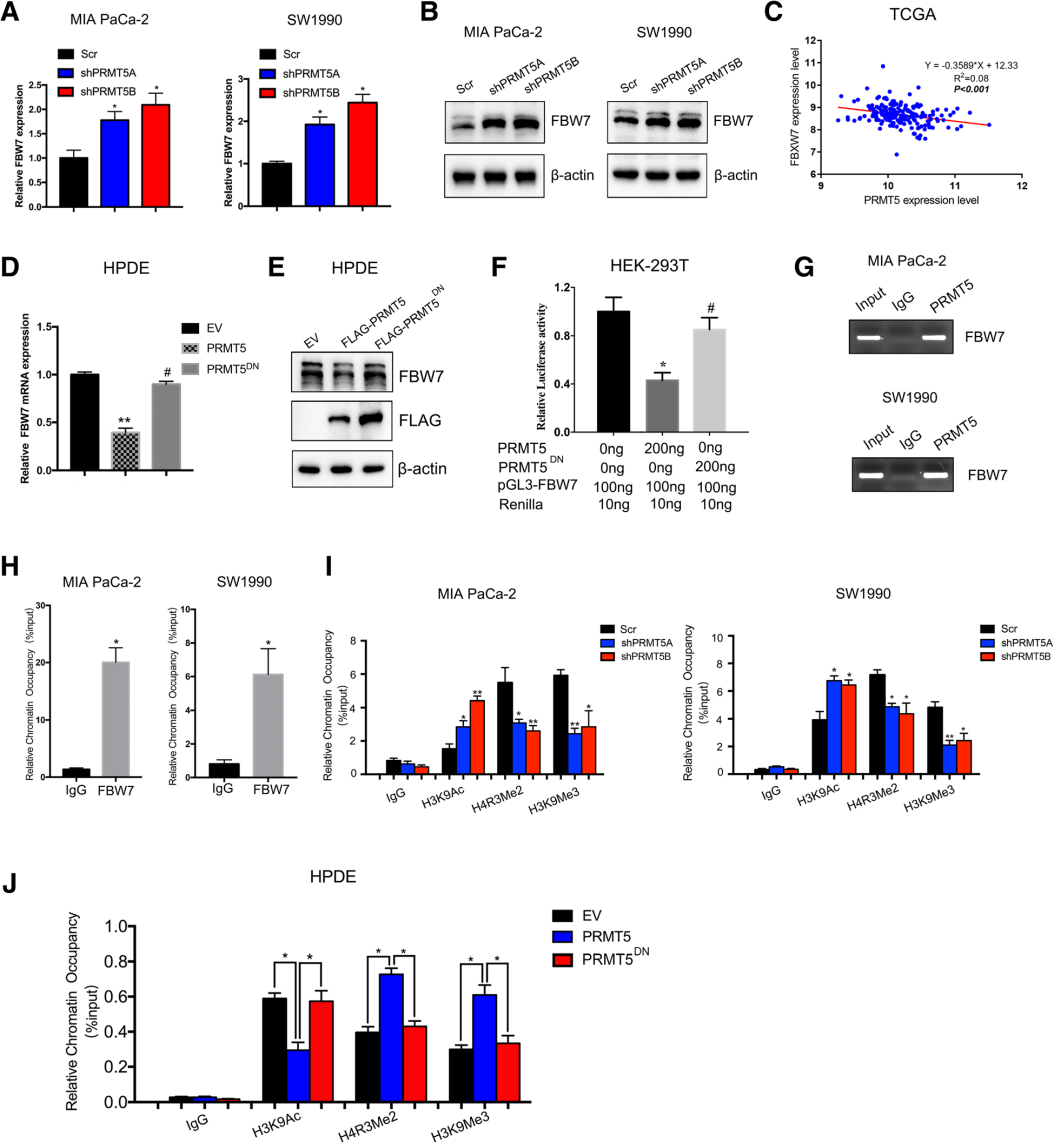
PRMT5-mediated epigenetic silencing of FBW7 leads to increased cMyc levels **a.** In PRMT5-silenced MIA PaCa-2 and SW1990 cells, the mRNA levels of FBW7 were increased. **b.** PRMT5 knockdown increased FBW7 protein levels. **c.** PRMT5 expression was negatively correlated with FBW7 expression in the TCGA-PAAD dataset of pancreatic cancer patients. **d.** In HPDE cells, overexpressing PRMT5 decreased FBW7 mRNA levels, but PRMT5^DN^ did not regulate FBW7 expression. **e.** Overexpressing PRMT5 in HPDE cells decreased FBW7 protein levels, but PRMT5^DN^ exerted no impact on FBW7 protein levels. **f.** The results of the dual luciferase assay in HEK-293 T cells showed that although PRMT5 suppressed FBW7 promoter activity, PRMT5^DN^ did not significantly regulate FBW7 promoter activity. **g-h.** The ChIP assay results demonstrated that PRMT5 occupied the promoter region enriched with CpG islands. **i.** PRMT5 knockdown decreased the occupancy of the heterochromatin markers H4R3me2 and H3K9me3, and the euchromatin marker H3K9ac, which reflects active transcription, was increased in MIA PaCa-2 and SW1990 cells with PRMT5 knockdown. **j**. Finally, we performed ChIP in HPDE cells with low PRMT5 expression. The ChIP results showed that PRMT5 increased the occupancy of heterochromatin markers such as H4MR3me2 and H3K9me3 and decreased that of the active chromatin marker H3K9ac decreased. However, the transferase-dead PRMT5^DN^ mutant had no such effect

FBW7 is the E3 ubiquitin ligase responsible for regulating cMyc stability and is frequently inactivated in cancers. Thus, we asked whether PRMT5 regulated cMyc stability through FBW7. PRMT5-silenced MIA PaCa-2 and SW1990 cells exhibited an increase in FBW7 mRNA and protein levels (Fig. 5a and b). Additionally, in the TCGA cohort, we observed a negative correlation between PRMT5 expression and FBW7 expression (Fig. 5c). Moreover, our results in HPDE cells demonstrated that wild-type PRMT5 could decrease FBW7 mRNA and protein levels, while the dominant-negative mutant of PRMT5 exerted no effect on FBW7 expression, suggesting that PRMT5 might regulate FBW7 at the transcriptional level (Fig. 5d and e). Therefore, we measured the impact of PRMT5 on FBW7 promoter activity; the dual-luciferase assay results demonstrated that although PRMT5 could suppress FBW7 promoter activity, the PRMT5 dominant-negative mutant played no significant role in the suppression of FBW7 promoter activity (Fig. 5f). By performing a ChIP assay, we demonstrated that PRMT5 could occupy the promoter region of FBW7, which was reported to be hypermethylated in cancer cells (Fig. 5g and h). Through a quantitative ChIP assay, we demonstrated that silencing PRMT5 expression in PRMT5-silenced MIA PaCa-2 and SW1990 cells decreased the occupancy of heterochromatin markers, including H4R3Me2 and H3K9Me3 on the PRMT5 substrate but increased the occupancy of the euchromatin marker H3K9ac (Fig. 5i). However, we overexpressed PRMT5 in PRMT5-low HPDE cells and then performed a ChIP assay. These results demonstrated that the introduction of wild-type PRMT5 increased the occupancy of heterochromatin markers such as H4R3me2 and H3K9me3 and decreased the occupancy of the euchromatin marker H3K9ac. However, the methyltransferase-dead mutant of PRMT5 exerted no such effect, further reinforcing the hypothesis that PRMT5 epigenetically regulates FBW7 expression (Fig. 5j). Collectively, these results suggest that PRMT5 could epigenetically suppress FBW7 expression in pancreatic cancer.

### PRMT5 regulates proliferation and aerobic glycolysis via the FBW7/cMyc axis

**Fig. 6:**
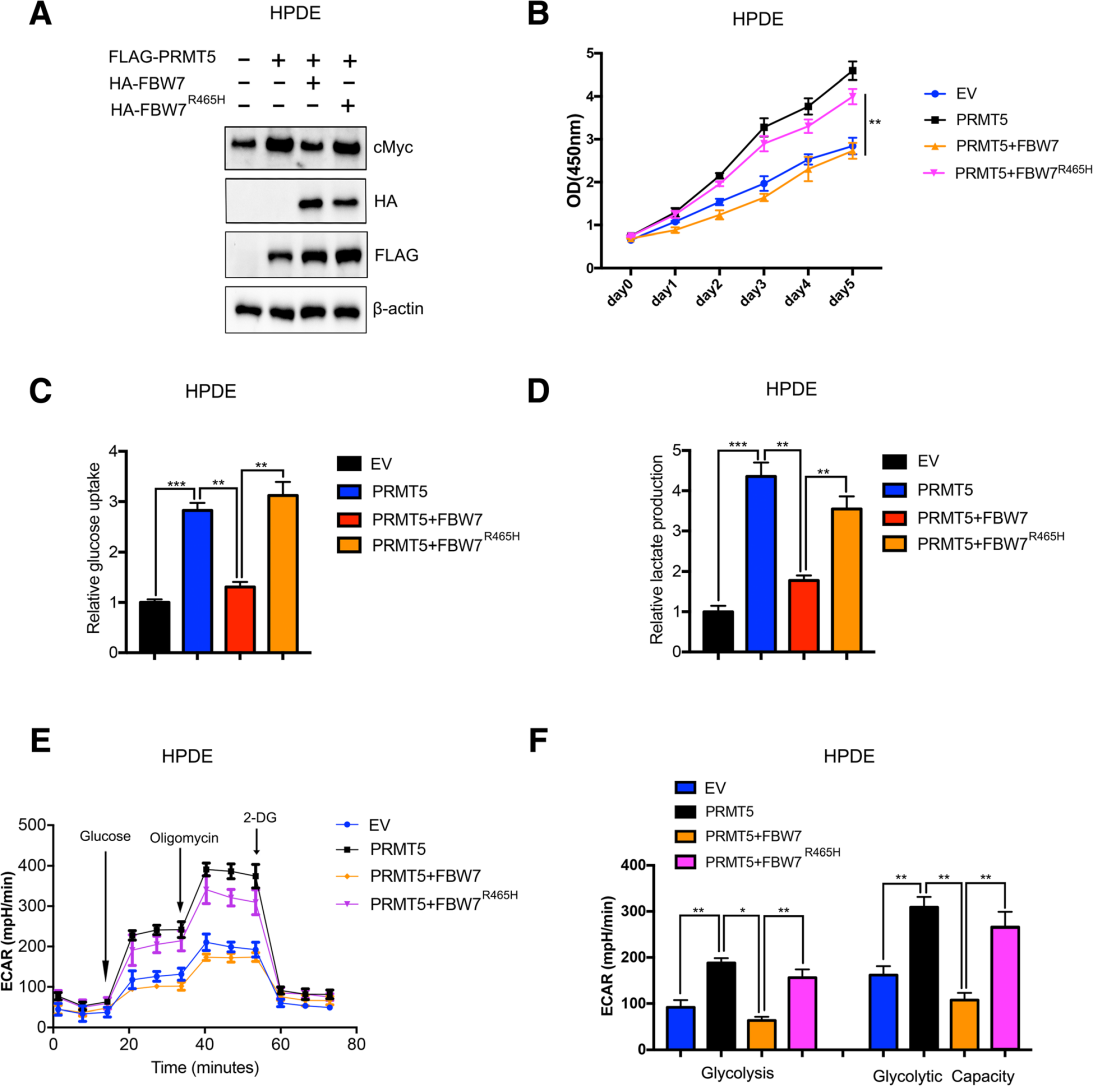
PRMT5 regulates proliferation and aerobic glycolysis via the FBW7/cMyc axis **a.** Overexpression of wild-type FBW7 in PRMT5-overexpressing HPDE cells attenuated the increase in the cMyc protein level, but the FBW7^R465H^ mutant had no such effect. **b.** The CCK-8 assay results demonstrated that wild-type FBW7 decreased the increase in cell viability caused by PRMT5, while the FBW7^R465H^ mutant, which lacked enzymatic activity, did not. **c.** FBW7 suppressed the increase in glucose uptake caused by PRMT5 in HPDE cells, while the FBW7^R465H^ mutant did not. **d.** FBW7 inhibited the increase in lactate production induced by PRMT5 in HPDE cells, but the FBW7^R465H^ mutant had little impact. **e-f.** The ECAR measurement results showed that FBW7 mitigated the increase in glycolysis and glycolytic capacity caused by PRMT5, but the FBW7^R465H^ mutant did not, suggesting that PRMT5 regulates aerobic glycolysis via the FBW7/cMyc axis

As discussed above, PRMT5 could epigenetically regulate FBW7 expression and stabilize cMyc at the protein level. We asked whether the impact of PRMT5 in regulating proliferation and aerobic glycolysis is mediated through the FBW7/cMyc axis. In HPDE cells, the introduction of wild-type FBW7 attenuated the increase in cMyc protein levels caused by PRMT5. However, cotransfection with PRMT5 and the FBW7^R465H^ mutant, which lacked ubiquitin ligase activity for controlling cMyc stability had no such effect, suggesting that PRMT5 could regulate cMyc via FBW7 (Fig. 6a). Moreover, the CCK-8 cell proliferation assay results demonstrated that FBW7 but not FBW7^R465H^ could mitigate the increase in cell viability caused by PRMT5, reinforcing the hypothesis that PRMT5 regulates cell proliferation via the FBW7/cMyc axis (Fig. 6b). In HPDE cells, overexpression of PRMT5 increased glucose uptake and lactate secretion, but cotransfection with PRMT5 and wild-type FBW7 attenuated the increases in glucose uptake and lactate secretion. However, cotransfection with PRMT5 and the FBW7^R465H^ mutant, which lacked the ability to control cMyc stability, had no such effect (Fig. 6c and d). Consistent with the results of the glucose intake and lactate secretion assays, the ECAR measurement results further validated that PRMT5 could regulate aerobic glycolysis via the FBW7/cMyc axis in HPDE cells (Fig. 6e and f). Collectively, these results suggest that PRMT5 regulates proliferation and aerobic glycolysis via the FBW7/cMyc axis.

**Fig. 7:**
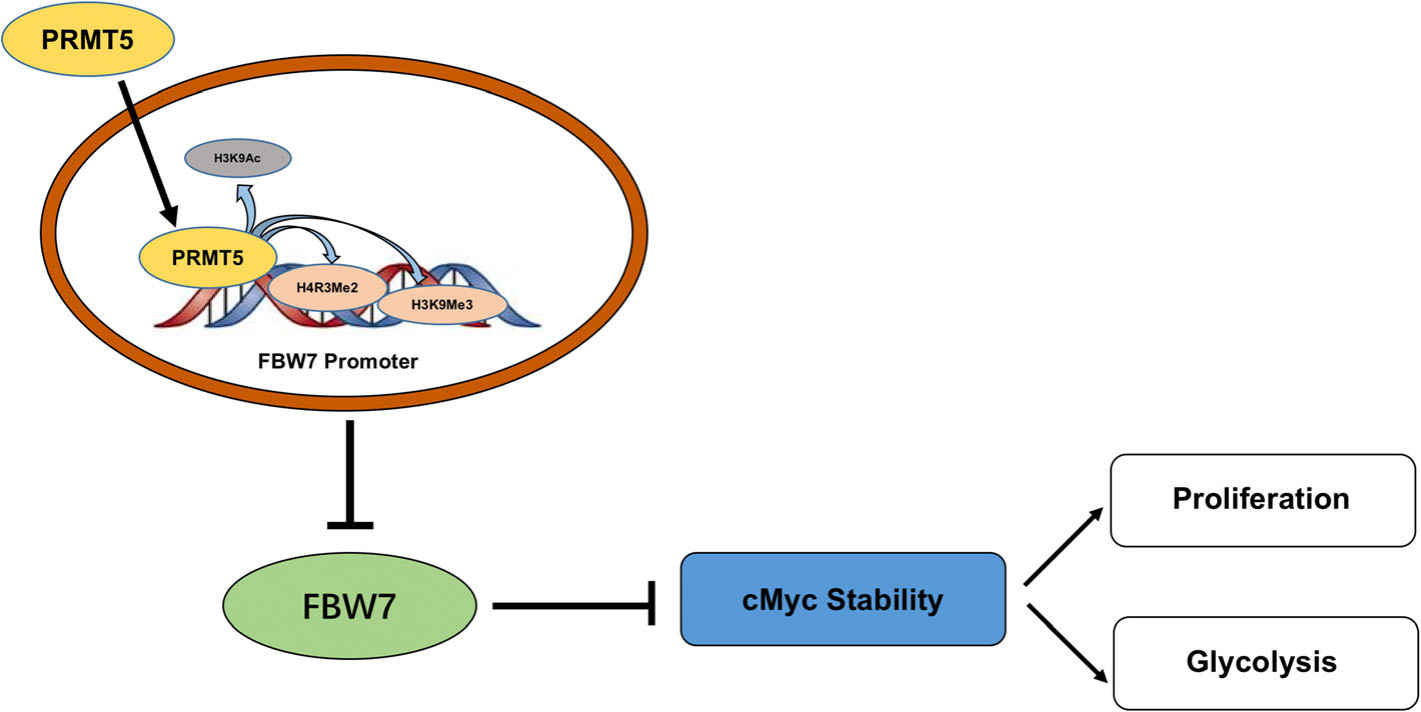
Schematic representation of the working model In pancreatic cancer, upregulated PRMT5 could epigenetically silence the expression of the E3 ubiquitin ligase FBW7, resulting in cMyc stabilization and a subsequent increase in the proliferation of and glycolysis in pancreatic cancer cells

In conclusion, we first reported that increased expression of PRMT5 is an unfavorable prognostic marker in pancreatic cancer. Furthermore, we demonstrated by in vitro and in vivo studies that PRMT5 could regulate tumorigenesis. Mechanistic explorations showed that PRMT5 could epigenetically inhibit the expression of the tumor suppressor gene FBW7, leading to increased expression of the cMyc oncogene at the protein level and the subsequent enhancement of aerobic glycolysis, which sustained the proliferation of pancreatic cancer cells (Fig. 7). Further research into the roles of the PRMT5/FBW7/cMyc axis might assist the development of novel prognostic and treatment targets in pancreatic cancer.

## Discussion

Protein arginine methylation catalyzed by PRMTs represents one of the histone modifications and epigenetically regulates multiple cellular processes. However, the physiological roles of PRMTs in pancreatic cancer have seldom been studied. In the present study, we uncovered novel roles for the PRMT family member PRMT5 in pancreatic cancer and demonstrated that PRMT5 expression could predict overall survival in pancreatic cancer. Mechanistic explorations revealed that PRMT5 could suppress the expression of the tumor suppressor gene FBW7, leading to stabilization of cMyc. Activation of the PRMT5/FBW7/cMyc axis ultimately contributed to enhanced aerobic glycolysis and the sustained proliferation of pancreatic cancer cells.

The role of PRMT5 in cancer has received increasing attention recently. PRMT5 regulates many cellular processes via its methyltransferase activity, and it specifically catalyzes the methylation of arginine residues in histone and nonhistone proteins. PRMT5 has emerged as an epigenetic enzyme that mainly represses the transcription of target genes via symmetric dimethylation of arginine residues such as H4R3, H3R8 and H2AR3 [33]. Accumulating evidence suggests that PRMT5 may function as an oncogene to drive cancer cell growth and metastasis by epigenetically silencing several tumor suppressor genes. For example, PRMT5 could modify H3R8 and H4R3 in the promoter region of the tumor suppressor Rb, leading to silencing of Rb expression and the subsequent proliferation of leukemia and lymphoma cells [34, 35]. PRMT5 could interact with bromodomain protein 7 and epigenetically silence the expression of suppressor of tumorigenicity 7 (ST7) via hypermethylation of H3R8 and H4R3 in the promoter region [36]. In lung cancer, PRMT5 could specifically catalyze the symmetrical dimethylation of histone H4R3 in the promoter regions of miR-99 family members, leading to increased growth and metastasis of lung cancer cells [13]. One mechanism that controls PRMT5-targeted gene expression is the methylation of histone H4R3 by PRMT5, which could recruit DNA methyltransferase 3A (DNMT3A), leading to DNA methylation and the formation of heterochromatin in the promoter region of its target genes [37]. PRMT5 could also target nonhistone substrates in cancer; for example, it can methylate arginine residues on the androgen receptor (AR) in prostate cancer and regulate prostate cancer malignancy [38, 39]. In hepatocellular carcinoma, symmetric dimethylation of R321 on sterol regulatory element-binding protein 1 (SREBP1) activates de novo lipogenesis and tumorigenesis [40]. In addition, the p65 subunit of the ubiquitous inducible transcription factor NF-κB can be modified by arginine methylation via PRMT5, thus regulating inflammatory responses and tumorigenesis [41]. Our studies are the first to identify the important roles of PRMT5 in predicting overall survival and promoting tumorigenesis in pancreatic cancer. Although pharmacological treatments directly targeting PRMT5 are not yet available, some Epizyme inhibitors such as EPZ015666 carry promise for utilization in cancer. In the reports, the authors performed MTT assays to examine the cytotoxicity of EPZ015666 on the cell lines that used. Even to a concentration of 5 μM, the inhibitor did not reduce the cell viability, suggesting that the inhibitory effects of EPZ01566 were not as a result of drug cytotoxicity [42]. Based on the important roles of PRMT5 in pancreatic cancer, trials to test the efficacy of these inhibitors might provide novel strategies for the treatment of pancreatic cancer.

Aberrant cancer cell metabolism has been regarded as one hallmark of cancer, and among the hallmarks, aerobic glycolysis has received increasing attention [43]. In pancreatic cancer, aerobic glycolysis not only provides nutrients for cell proliferation but also regulates metastasis, immune evasion, chemotherapy and radiotherapy resistance. In pancreatic cancer, glycolysis is considered a promising target, and an in-depth search for molecules that regulate aerobic glycolysis might aid the discovery of novel strategies [44]. Epigenetic factors and aerobic glycolysis are closely related to each other [45]. For example, Sirtuin family members are histone deacetylases and can regulate glucose and glutamine metabolism [46]. In addition, histone lysine methyltransferases and demethylases could regulate aerobic glycolysis. In breast cancer, the histone methyltransferase Set8 could regulate aerobic glycolysis via stabilizing HIF1α [47]. In glioblastoma, PRMT5 could regulate PTEN expression and induce the activation of Akt and ERK, components of a pathway that could regulate aerobic glycolysis [48, 49]. In lung cancer, PRMT5 has been reported to regulate the HIF1 signaling pathway, but its direct roles in aerobic glycolysis under the control of the HIF1 signaling pathway have seldom been reported [50, 51]. Our results demonstrated that PRMT5 could regulate aerobic glycolysis in pancreatic cancer via cMyc instead of HIF1α. However, we believe that the impact of PRMT5 on aerobic glycolysis is not restricted to its impact on cMyc protein stability. For example, PRMT5 has been reported to associate with cMyc, and PRMT5 has been reported to catalyze the methylation or dimethylation of H3R2, leading to active transcription [52]. Therefore, cMyc may recruit PRMT5 to its downstream glycolytic genes, such as glucose transporter 1 (GLUT1) and hexokinase 2 (HK2), and methylate H3R2 on these genes, leading to active transcription of glycolytic genes and promotion of the glycolysis process [53]. Moreover, PRMT5 might directly catalyze the methylation of arginine residues on glycolytic or glycolysis-related proteins. For example, p53 is a substrate of PRMT5 and plays important roles in aerobic glycolysis in cancer. The glycolytic protein enolase-1 (ENO1) has been reported to be methylated by PRMT5, but the effect of ENO1 methylation on aerobic glycolysis needs further investigation [54].

In addition to mutational inactivation, transcriptional, translational and posttranslational approaches have been used to control the intracellular levels of FBW7. The tumor suppressor p53 has been shown to regulate FBW7 transcription, and the first exon of FBW7 contains a p53 binding site [55]. The transcription factor Hes5 (hairy enhancer of split 5) could bind the promoter region of FBW7 and inhibit expression [56]. Some microRNAs, such as miR223, miR25 and miR129-5p, can suppress the translation of FBW7. Moreover, FBW7 activity and protein levels can be controlled posttranslationally by enzymes such as the ERK kinase, PKC (protein kinase C), PIN1 (peptidylprolyl cis/trans isomerase, NIMA-interacting protein 1) and the deubiquitinase USP9X (ubiquitin specific protease 9X) [57, 58]. However, the epigenetic mechanisms linking DNA methylation and histone methylation and acetylation to FBW7 expression in pancreatic cancer have seldom been reported. In breast cancer, promoter hypermethylation leads to the inactivation of FBW7. The present study is the first to demonstrate that PRMT5 activity could lead to FBW7 expression via chromatin modification in the FBW7 promoter region. Further investigations are needed to identify the transcription factors that could recruit PRMT5 to the FBW7 promoter region. FBW7 is an important tumor suppressor and has been reported to regulate many physiological processes, such as chemotherapy resistance, which is a challenge in improving the prognosis of pancreatic cancer [59]. Therefore, it is necessary to conduct an in-depth study to reveal the roles of PRMT5 in FBW7-related processes with an aim to discover novel strategies for the treatment of pancreatic cancer.

In conclusion, we identified PRMT5 as a novel marker for predicting the prognosis of pancreatic cancer and reported its novel role in regulating aerobic glycolysis via the FBW7/cMyc axis. Further investigations to determine the roles of PRMT5 in pancreatic cancer might aid the discovery of novel therapeutic targets and improve the prognosis of pancreatic cancer.

## Conclusions

In summary, the present study revealed PRMT5 as a novel prognostic marker for overall survival in pancreatic cancer. Mechanistic studies demonstrated that PRMT5 could epigenetically suppress FBW7 expression and elevate cMyc stability, leading to tumorigenicity and aerobic glycolysis in pancreatic cancer. The results of this study might provide novel treatment strategies for pancreatic cancer.

## A. Additional file

Additional file 1: **Table S1.** Primers sequences used in the text. **Table S2.** Clinicopathological features and correlation of PRMT5 expression in pancreatic ductal adenocarcinoma. **Table S3.** Basic features of pancreatic cancer patients in TCGA database. (DOCX 24 kb)

## Abbreviations

ChIP: Chromatin immunoprecipitation
DFS: Disease-free survival
ECAR: Extracellular acidification rate
FBW7: F-box and WD repeat domain-containing 7
FDG: Fludeoxyglucose
OS: Overall survival
PET/CT: Positron emission tomography and computed tomography
PRMT5: Protein arginine methyltransferase 5
