# Supplementary material for: PRMT5 enhances tumorigenicity and glycolysis in pancreatic cancer via the FBW7/cMyc axis
Source: Cell Commun Signal. 2019 Mar 29;17:30. doi: 10.1186/s12964-019-0344-4 (PMC6440122; doi:10.1186/s12964-019-0344-4)
Supplement: Supplementary file 1 — Table S1. Primers sequences used in the text. Table S2. Clinicopathological features and correlation of PRMT5 expression in pancreatic ductal adenocarcinoma. Table S3. Basic features of pancreatic cancer patients in TCGA database. (DOCX 24 kb) [file 12964_2019_344_MOESM1_ESM.docx]

**Additional file 1: Table S1. Primers sequences used in the text**

| PRMT5 forward | 5’- CACCTTCAGCCATCCCAACAGAG-3’ |
| --- | --- |
| PRMT5 reverse | 5’- CCATGAGAACATCCCAGGAGAGTG-3’ |
| FBW7 forward | 5’- CCACTGGGCTTGTACCATGTT-3’ |
| FBW7 reverse | 5’- CAGATGTAATTCGGCGTCGTT-3’ |
| cMyc forward | 5’-AGAGTCTGGATCACCTTCTGCTGG -3’ |
| cMyc reverse | 5’- CGGTTGTTGCTGATCTGTCTCAGG -3’ |
| β-actin forward | 5’- CCTGACGGCCAGGTCATCACCAT -3’ |
| β-actin reverse | 5’- ACGGAGTACTTGCGCTCAGGAGGA -3’ |

| **Table S2. Clinicopathological features and correlation of PRMT5 expression in pancreatic ductal adenocarcinoma** | | | | |
| --- | --- | --- | --- | --- |
|  |  | PRMT5-High | PRMT5-Low |  |
| Characteristics | No. | score( ++/+++)(n=37) | score(-/+)(n=18) | P Value |
| Age(y) |  |  |  |  |
| <60 | 22 | 14 | 8 | 0.64 |
| ≥60 | 33 | 23 | 10 |  |
| Gendar |  |  |  |  |
| Female | 23 | 13 | 10 | 0.15 |
| Male | 32 | 24 | 8 |  |
| Tumor size(cm) |  |  |  |  |
| <4.0 | 34 | 21 | 13 | 0.27 |
| ≥4.0 | 21 | 16 | 5 |  |
| Tumor differentiation |  |  |  |  |
| Well | 10 | 6 | 4 | 0.23 |
| Moderate | 31 | 19 | 12 |  |
| Poor | 14 | 12 | 2 |  |
| Lymph node status(stage) |  |  |  |  |
| Negative(ⅡA) | 35 | 24 | 11 | 0.79 |
| Positive(ⅡB) | 20 | 13 | 7 |  |
| Vessel Infiltration |  |  |  |  |
| Negative | 43 | 28 | 15 | 0.52 |
| Positive | 12 | 9 | 3 |  |
| Nerve Infiltration |  |  |  |  |
| Negative | 13 | 8 | 5 | 0.61 |
| Positive | 42 | 29 | 13 |  |
| Median survival  (in months) |  |  |  |  |
| P values were derived with Pearson chi-square tests. | | | | |
| P values was derived with log rank test. | | | | |
| All statistical tests are two sided. | | | | |
| Abbreviations: PRMT5-- Protein Arginine Methyltransferase 5 | | | | |

**Table S3. Basic features of pancreatic cancer patients in TCGA database.**

|  | **PRMT5** | | | |  |
| --- | --- | --- | --- | --- | --- |
|  | **low level** | | **high level** | | P |
|  | N | % | N | % |  |
| **Age (median, IQR)** | 65(56-72) | | 65(57-75) | | 0.746 |
| **Sex** |  |  |  |  | 0.33 |
| male | 43 | 48.9% | 37 | 41.6% |  |
| female | 45 | 51.1% | 52 | 58.4% |  |
| **Primary Tumor Site** |  |  |  |  | 0.329 |
| Body | 7 | 8.0% | 7 | 7.9% |  |
| Head | 68 | 77.3% | 70 | 78.7% |  |
| Tail | 5 | 5.7% | 9 | 10.1% |  |
| Other | 8 | 9.1% | 3 | 3.4% |  |
| **Histologic Type** |  |  |  |  | 0.003 |
| Ductal Adenocarcinoma | 66 | 75.0% | 81 | 91.0% |  |
| Adenocarcinoma other type | 22 | 25.0% | 7 | 7.9% |  |
| Undifferentiated | 0 | 0.0% | 1 | 1.1% |  |
| **AJCC Stage** |  |  |  |  | 0.379 |
| 0 | 2 | 2.3% | 1 | 1.1% |  |
| I | 14 | 15.9% | 7 | 7.9% |  |
| II | 68 | 77.3% | 78 | 87.6% |  |
| III | 1 | 1.1% | 2 | 2.2% |  |
| IV | 3 | 3.4% | 1 | 1.1% |  |

Abbreviations: AJCC—American Joint Committee on Cancer
